# Decoding the Time Course of Spatial Information from Spiking and Local Field Potential Activities in the Superior Colliculus

**DOI:** 10.1523/ENEURO.0347-22.2022

**Published:** 2022-11-30

**Authors:** Michelle R. Heusser, Clara Bourrelly, Neeraj J. Gandhi

**Affiliations:** 1 Department of Bioengineering, University of Pittsburgh, Pittsburgh, PA 15213; 2 Center for Neural Basis of Cognition (CNBC), University of Pittsburgh, Pittsburgh, PA 15213; 3 Department of Neuroscience, University of Pittsburgh, Pittsburgh, PA 15213

**Keywords:** brain-computer interface, decoding, eye movement, frontal eye field, oculomotor, sensorimotor transformation

## Abstract

Place code representation is ubiquitous in circuits that encode spatial parameters. For visually guided eye movements, neurons in many brain regions emit spikes when a stimulus is presented in their receptive fields and/or when a movement is directed into their movement fields. Crucially, individual neurons respond for a broad range of directions or eccentricities away from the optimal vector, making it difficult to decode the stimulus location or the saccade vector from each cell’s activity. We investigated whether it is possible to decode the spatial parameter with a population-level analysis, even when the optimal vectors are similar across neurons. Spiking activity and local field potentials (LFPs) in the superior colliculus (SC) were recorded with a laminar probe as monkeys performed a delayed saccade task to one of eight targets radially equidistant in direction. A classifier was applied offline to decode the spatial configuration as the trial progresses from sensation to action. For spiking activity, decoding performance across all eight directions was highest during the visual and motor epochs and lower but well above chance during the delay period. Classification performance followed a similar pattern for LFP activity too, except the performance during the delay period was limited mostly to the preferred direction. Increasing the number of neurons in the population consistently increased classifier performance for both modalities. Overall, this study demonstrates the power of population activity for decoding spatial information not possible from individual neurons.

## Significance Statement

We make countless goal-directed eye movements each day. Individual neurons that signal for the appearance of a visual stimulus and/or the execution of a rapid eye movement often fire at comparable levels for very different spatial parameters. We recorded both spiking activity and local field potential (LFP) signals across many channels simultaneously and asked whether the spatial parameter of target or saccade direction can be decoded across a broad range of the visual field. Applying simple categorical classifiers to “populations” of neurons, we found that both spiking and LFP activity were informative of direction early on, starting at the initial visual response and continuing through movement initiation. This investigation demonstrates the advantage of a population-level framework over traditional approaches.

## Introduction

**Figure 1.**
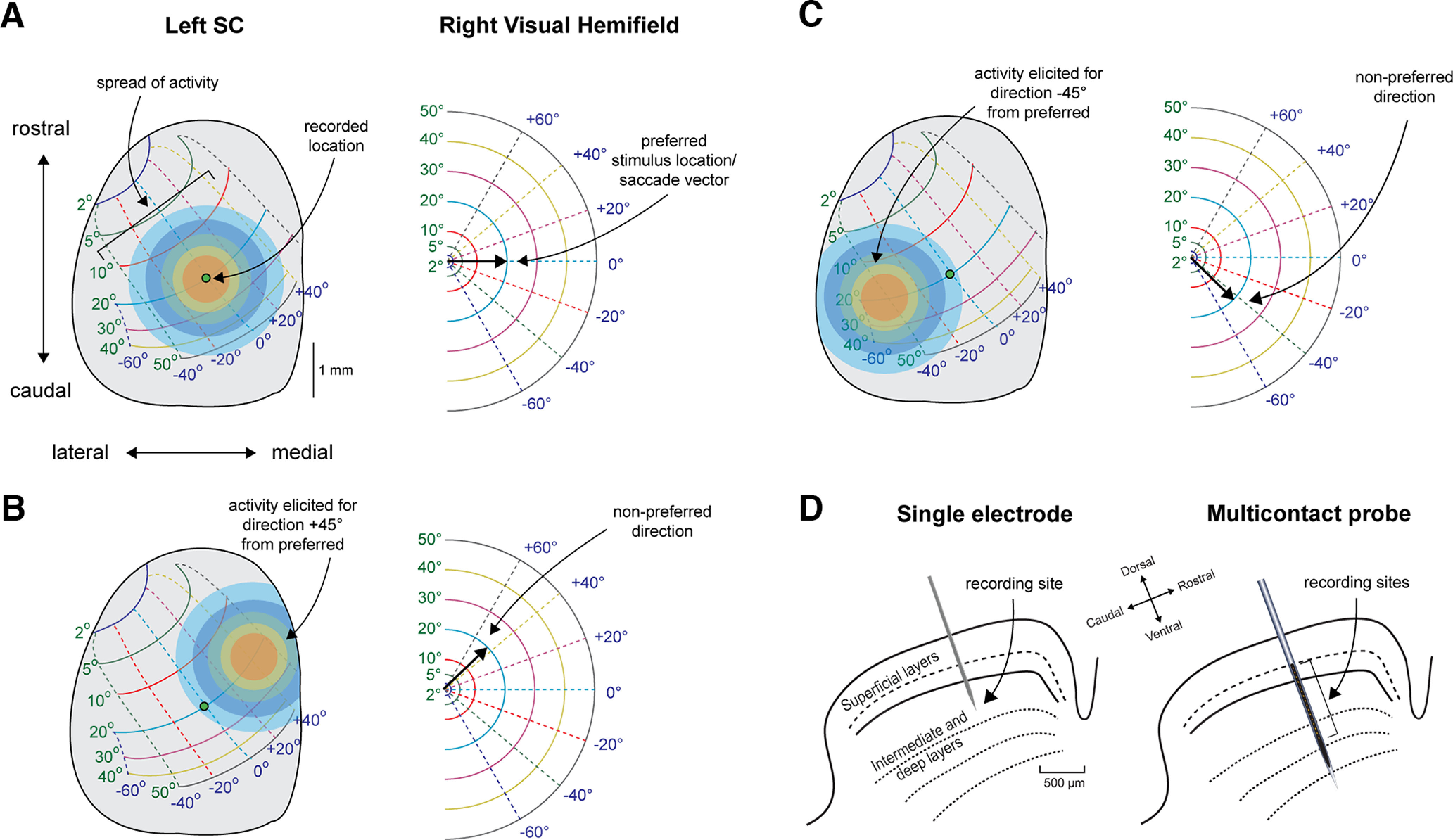
Schematic of spiking properties of recorded SC neurons. ***A***, A widely accepted model of the left SC topographic map and the corresponding right visual hemifield. When a visual stimulus is presented in a particular location and/or a saccade is made to that location, neurons are active across the SC map. The hot spot of activity for the example recorded location is at the green dot when the amplitude and direction of the stimulus/saccade are 20° and 0°, respectively; activity spreads spatially across the SC in a Gaussian-like manner. ***B***, ***C***, SC activity elicited for vectors 45° (***B***) and −45° (***C***) away from the preferred direction of the recorded neuron. These hypothetical cases highlight how two very different direction vectors can elicit similar activity levels at the recorded location. Figure panels ***A*** through ***C*** adapted from Gandhi and Katnani (2011). The same conceptual quandary remains even if the topographic map is updated to reflect unequal representations of upper and lower hemifields (Hafed and Chen, 2016). ***D***, Traditional single electrode approach into the SC (left) compared with an advanced recording technique with a multichannel laminar probe (right). In both cases, the insertion angle is orthogonal to the SC surface, yielding neuron(s) at only one location on the SC map (e.g., the location of the green dot in the previous panels). Figure panel adapted from Jagadisan and Gandhi (2022).

We interact with our environment by redirecting our line of sight to objects of interest. A large network of neural structures is involved in this process of sensorimotor integration. The superior colliculus (SC), a topographically organized laminar structure in the subcortex, is essential for the control of visually guided eye movements known as saccades (for review, see Gandhi and Katnani, 2011; Basso and May, 2017). Neurons in the superficial layers are primarily sensory, producing a burst of spikes when a stimulus is presented in their receptive fields. Neurons in the deep layers are predominantly motoric, emitting a volley of spikes before the generation of a saccade in their movement field. Neurons in the intermediate layers exhibit both sensory and motor bursts. In reality, the extent of visual and motor bursts varies inversely as a continuum along the dorsoventral axis (Mohler and Wurtz, 1976; Ikeda et al., 2015; Massot et al., 2019; Jagadisan and Gandhi, 2022). Within a layer, neurons vary in their preferred vector along the mediolateral and rostral-caudal axes, respectively. An individual neuron at a particular position on the SC map will exhibit maximal activity for its optimal vector (in sensory and/or motor domains) and gradually less for vectors away from it (Goldberg and Wurtz, 1972; Wurtz and Goldberg, 1972; Sparks, 1975; Sparks et al., 1976). Take, for example, a hypothetical deep layer neuron recorded at the location on the SC map shown in Figure 1*A* as a green dot. If the executed saccade is horizontal and rightward with a 20° amplitude (Fig. 1*A*, right panel), this neuron will be located at the “hot spot” of activity produced in the SC. The farther neurons are from this hot spot, the less active they will be, as the spread of activity is thought to decay in a Gaussian-like manner. Now suppose the amplitude of the executed saccade is held constant but the direction is at an angle 45° counterclockwise (Fig. 1*B*). In this case, the neuron will no longer be located at the hot spot on the SC map and thus will have much less activity. One can imagine a case in which the recorded neuron has similar activity levels for yet other saccade vectors (e.g., Fig. 1*C*). In such conditions, the activity elicited at the recorded location is similar, so it follows that discriminating the direction of saccade vectors far away from the preferred direction of a single neuron is challenging.

Experimenters most often approach the SC with probes inserted orthogonally to the SC surface. The typical approach for isolating one neuron and recording its activity during behavioral tasks is represented in the left panel of Figure 1*D*. However, with recent technological advances, researchers can record from small “populations” of neurons via a laminar probe, with electrode contacts spanning the dorsoventral axis of the SC (Fig. 1*D*, right panel). Neurons along this axis systematically vary in the degree to which they signal for sensory and motor parameters across depth (Ikeda et al., 2015; Massot et al., 2019; Mohler and Wurtz, 1976) but are thought to encode roughly the same intended stimulus location/saccade vector, thus yielding a highly homogenous population. In contrast, placing multicontact electrodes into cortical oculomotor structures such as the frontal eye fields (FEF) yields a heterogenous population; each neuron in the recorded population will signal maximally for a very different amplitude and direction of the intended eye movement (Bruce et al., 1985; Sommer and Wurtz, 2000). In such structures, it is easier to appreciate how recording from populations of neurons would provide an advantage in discriminating spatial parameters of the stimulus or saccade vector (see, for example, the classic idea of population vector averaging in Georgopoulos et al., 1983; also see Graf and Andersen, 2014; Jia et al., 2017). Given that the topography of the SC does not provide the same spatial variability when using the standard electrode approach, it is not as intuitive that population activity could improve discriminability of spatial parameters across a broad range of the visual field over that of single units.

We challenged this notion by testing whether information about a broad range of visual stimulus locations and/or saccade directions can be obtained from activities of small populations of simultaneously recorded SC neurons within a specific location on the SC topographic map, and if so, at what time(s) during the sensorimotor integration process this spatial information is present. Accordingly, we investigated the time course of direction discriminability present in the spiking activity of SC neural populations and compared the neurons’ encoding properties with a second signal modality, the local field potential (LFP), which at a given recording site reflects the aggregate activity of nearby neurons through a measure of their extracellular voltage (Buzsáki et al., 2012). We recorded both signals simultaneously across layers of the SC while rhesus monkeys (*Macaca mulatta*) performed delayed saccades to one of eight targets radially equidistant in direction. We then trained a simple linear classifier to output the most likely direction to which small windows of spiking or LFP activity belonged. The performance of the classifier across time and directions gives a comprehensive indication of the spatial encoding properties of SC activity during sensorimotor integration. We found that both spiking activity and LFPs from a small number of neurons can decode among the categories, including for the opposite hemifield, as early as the visual response.

## Materials and Methods

### Animal preparation

Two adult male rhesus monkeys (*M. mulatta*; BL and SU) were used in this study. All experimental procedures were approved by the University of Pittsburgh Institutional Animal Care and Use Committee. A sterile surgery was performed on each animal to implant a stainless-steel recording chamber (Narishige) angled 40° posterior with respect to vertical. Electrode penetrations through this chamber approach the SC orthogonal to its surface and traverse its dorsoventral axis along a track where neurons have similar response fields. Both animals were fitted with a thermoplastic mask to achieve fixation of the head during experimental sessions (Drucker et al., 2015).

### Data collection

**Figure 2.**
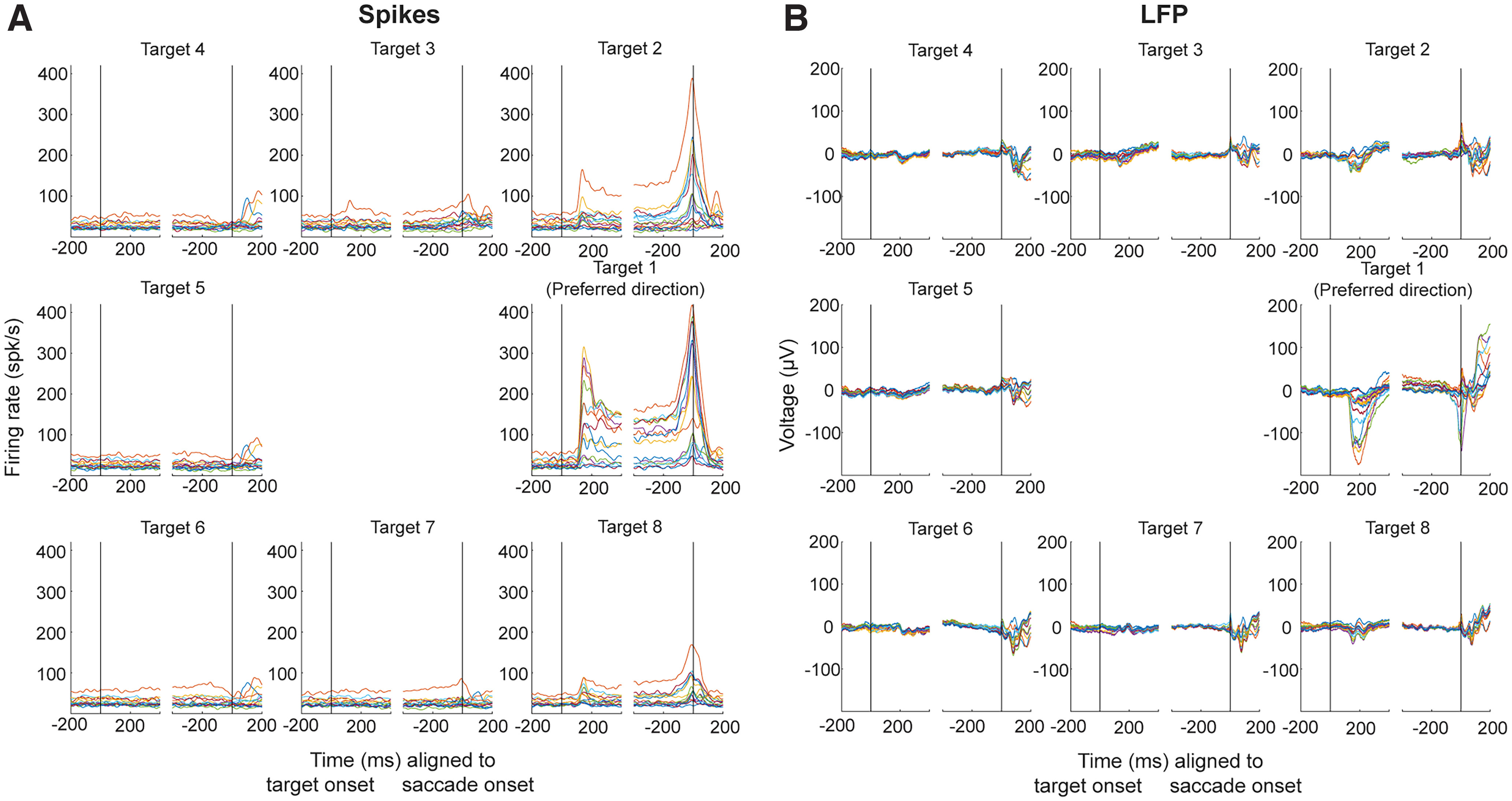
Peri-event values of spiking activity and LFPs simultaneously recorded across channels. ***A***, The across-trials mean firing rates for all 15 functional channels recorded during an example session are plotted aligned to target onset (left) and saccade onset (right) to eight radially equidistant targets. Each colored trace represents the spiking activity on one channel averaged across all trials to a particular target. Subplots are rotated so that the preferred target direction of this population is displayed horizontal and rightward with respect to center. ***B***, The across-trials mean LFP voltage values for all 15 channels are plotted using the same conventions as the spiking activity data.

Comprehensive details about neurophysiology and microstimulation are provided in Massot et al. (2019). In brief, a 16-channel (monkey BL) or 24-channel (SU) laminar microelectrode (Alpha Omega or Plexon, respectively) was inserted acutely into the SC to record neural activity across different layers. We stopped driving the electrode when characteristic SC spiking activity (typically visual and motor bursts) was qualitatively observed across many of the central-most recording channels. Then, some individual channels were stimulated (400 Hz, 20–40 μA, 100-200 ms; biphasic pulses, 200 μs pulse duration, 17 μs inter-pulse duration) to qualitatively determine an average evoked saccade vector, which was used as the preferred location for that session’s neural population. The raw activity recorded on each channel was separated into spike times (high pass filtered at 250 Hz and discretized using a standard threshold) and LFP (low pass filtered at 250 Hz). The majority of channels with task-related spiking activity were visuomotor neurons that exhibited large transient bursts both in response to a visual stimulus and before/during saccade. Only channels with peak spiking activity >20 spikes/s above baseline during either the visual or motor epochs were counted as functional channels and included in analyses (with total channels, U, ranging from 6 to 17 across sessions). For visualization only, spike counts were converted into firing rates by convolving each channel’s spike train with a Gaussian kernel of 10-ms width (as in Fig. 2*A*) and LFPs were bandpass filtered between 0.5 and 250 Hz with a notch filter at 60 Hz (as in Fig. 2*B*). Data from 15 sessions from monkey SU and three sessions from monkey BL were collected ( N=18 total sessions).

### Behavioral paradigm

Each monkey was trained to sit in a primate chair and perform a standard eye movement task in a dimly lit room. Eye position was tracked with an infrared eye tracker (EyeLink 1000, SR Research; see Massot et al., 2019 for additional details). During each recording session, animals performed many trials of a center-out delayed saccade task to one of eight possible targets evenly spaced in 45° increments around the fixation point. The delay period length was randomized from trial to trial, spanning 600–1200 ms (monkey BL) or 700–1500 ms (monkey SU). Each target had an equal likelihood of presentation, and “Target 1” was either placed at the spatial location corresponding to the estimated preferred saccade vector (for the majority of sessions) or at the position (10°, 0°) in polar coordinates. In the latter case, preferred target direction was re-defined as Target 1 following examination of the average spiking activity profiles for that session (as in Fig. 2). For sessions in which the target position was rotated and scaled, the circular mean direction of Target 1 was 131.6°, with mean amplitude of 14.6° ( N=12 sessions). The animal was given a liquid reward after executing a saccade to a location within 2° of the target position, and only these successful trials were included in analyses (typically yielding over 1000 total trials across all target directions per session).

### Classification methods

**Figure 3.**
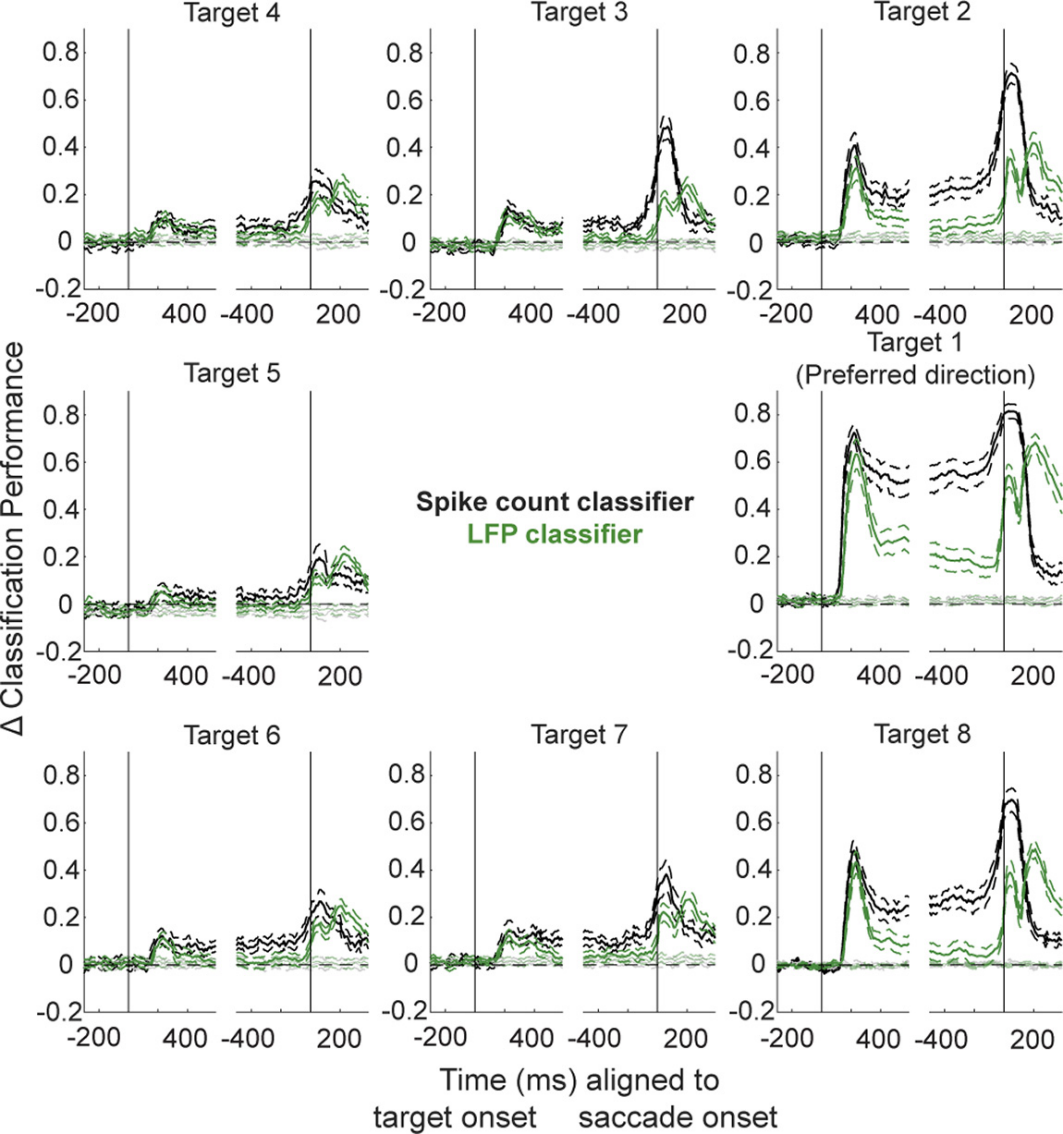
Linear discriminant classification of spiking and LFP activity. Sliding 100-ms windows of summed spike counts or average LFP voltage on each channel were used to train a linear discriminant analysis (LDA) model and test its ability to decode target direction. Mean (±SEM) across-session classifier performances for the spike count (black traces) and LFP classifiers (green traces) are plotted separately for each of eight target directions and aligned to target onset (left panels) or saccade onset (right panels). Chance level classifier performance was obtained by using shuffled class labels during the training phase. Performance values were grouped across sessions by aligning to each session’s preferred target direction (visualized here as the right middle panels), and the performance for each session and each target was baseline-subtracted before averaging. Values for each window are plotted aligned to the end of that window (e.g., performance of the classifier trained and tested on the 0- to 100-ms window following target onset is plotted at 100 ms on the *x*-axis).

Custom MATLAB code (MathWorks) was used for all analyses unless otherwise specified. Target location was decoded offline from population activity on each session individually. Summed spike count or average LFP voltage on each channel in 100-ms time windows, sliding in 10-ms increments across the duration of each individual trial, was labeled as belonging to Target 1 through Target 8 depending on the target location presented on that trial. For each individual 100-ms time bin, a separate linear discriminant classifier was trained on these summed spike counts or average LFP voltage values from a randomly selected 70% of total trials (pooled across all targets), and its performance was tested on the remaining 30%. Classifier performance was measured through the F1 score, a common metric for multiclass classifiers that takes into account both sensitivity and precision of the model for each target while countering any overfitting/underfitting to activity belonging to a particular target (Zhi et al., 2018). This process of randomly selecting 70% and 30% as training and test trials, respectively, was repeated for a total of 10 times for each window and each session to obtain an average classifier performance across iterations. Importantly, each classifier was trained and tested only on activity belonging to a particular time range and had no information about future or past windows that would influence performance within a given window. To determine an experimental chance level, target labels were randomly shuffled and the classification process described above was repeated. The actual chance level tended to closely match theoretical chance level performance of one out of eight targets, or 12.5% (results not shown). Before averaging across sessions, the classifier performance values of true and shuffled data in each window for each target were subtracted by the mean performance value for that target during the first 200 ms of the baseline period (i.e., 400–200 ms before target onset). This was done to normalize all sessions’ performance values as a change in performance relative to baseline. In all visualizations of classifier performance across time (Figs. 3, 5, and 6), values are plotted in a causal manner; for example, performance for the set of observations in the time window 100–200 ms after target onset is plotted at the 200-ms mark to represent that only historical activity was used to create and test a model of spatial location information.

A linear discriminant analysis (LDA) classifier is a supervised, geometric model that finds a hyperplane that maximally separates the input features between two categories, or “classes,” during the training phase. In this paradigm, there are U input features that correspond to the spiking or LFP activity on all functional channels (as described above, see Data collection), and there are 8 classes that correspond to the eight targets presented. Since an LDA model is by definition a binary classifier, we implemented a common technique called error-correcting output codes (ECOC) that fits a series of binary LDA classifiers in a one-versus-one manner to convert the model into a multiclass classifier, allowing for simultaneous classification into more than two categories (Derya Übeyli, 2008). During the testing phase, new data are shown to the model, and the class (i.e., target to which the activity corresponds) is determined by the position relative to the hyperplanes that were found during the training phase. To note, a pseudolinear discriminant classifier was implemented for spiking activity to combat the low or absent spike counts on some channels in certain time windows, which often leads to zero variance across observations and disrupts model fitting. To ensure that our results were robust to the type of classifier used, we also repeated all analyses using a ECOC support vector machine (SVM) algorithm and found classifier performance dynamics for both spiking and LFP activity to be quite similar to those found via ECOC LDA classification.

For analysis of the effect of window length on classifier performance, spike counts were summed and LFP voltages were averaged across each window of length [20, 50, 100, 200, 300] ms, which again were calculated in sliding increments of 10 ms (Fig. 5). Separately, the total number of functional channels recorded in a given session (see above, Data collection, for description of U, the total number of channels with task-related activity) were randomly shuffled and a subset was selected to be included as the input features to the spike count classifiers. This process was repeated for population sizes starting at 1 (equivalent to a single channel) and ending at U (Fig. 6*A*). The same randomly selected channels were used for the LFP classifiers (Fig. 6*B*).

To represent the spatial tuning properties of our neural populations during the many epochs of this behavioral task, we defined a range of times for each of five epochs (baseline, visual, early delay, late delay, and motor) during which we pulled out a single across-session classifier performance value for each target direction. Baseline performance was taken as the mean value in the range of 400–200 ms before target onset. Visual performance was taken as the maximum value around the time of the visual burst, typically occurring within the 100- to 200-ms range after target onset. Early delay performance was taken as the mean value in the range of 250–450 ms after target onset. Late delay performance was taken as the mean value in the range of 300–100 ms before saccade onset. Motor performance was taken as the maximum value around the time of the motor burst, typically occurring near saccade onset.

All statistical comparisons of classifier performance between signal modalities or epochs (in Figs. 8, 9) were performed using a paired two-tailed *t* test with *p* < 0.05 indicating a significant difference between the two distributions included in the comparison.

## Results

We set out to determine whether and at what times during a behavioral task do neural populations in a single column of the SC encode information about the spatial configuration of the trial. We employed a simple offline decoding algorithm (linear discriminant classifier) as a proxy for discriminability of spatial location (i.e., to which out of eight possible targets will an animal make a saccade on a given trial) during independent sliding windows of time throughout a behavioral task. This decoding algorithm was applied separately to the spiking activity of simultaneously recorded neurons and to the local field potential (LFP) recorded at the same locations across the dorsoventral axis of the nonhuman primate SC. Importantly, we remain agnostic with respect to whether the population encodes sensory and/or motor information at any given time. Instead, we will use any combination of terms “target/saccade location/direction” throughout the text and do not make any attempts to distinguish whether the spatial information being encoded is related to sensory (i.e., visual stimulus angle relative to eye position at fixation) or motor (i.e., intended saccade direction relative to starting eye position) representations.

In Figure 2*A*, the trial-averaged firing rates across all functional channels of an example session are plotted as colored traces aligned to target onset (left panels) and saccade onset (right panels). In general, the firing rates of these neurons are highest in the preferred direction (i.e., Target 1) and are less vigorous as the angular direction of the target/saccade moves away. In the opposite hemifield (i.e., Targets 4–6), activity across all channels is minimal. Figure 2*B* shows the trial-averaged voltage values of the LFPs across the same functional channels of the example session. Only minimal deflections from baseline levels are present for all target locations away from the preferred direction. Despite similar firing rate properties and LFP voltage deflection characteristics across all channels, can a method that utilizes the activity pattern across the population aid us in understanding how the SC encodes the spatial parameter of direction? To do this, we trained and tested simple linear classifiers to output the category (one of eight directions) to which either spiking or LFP activity belongs.

Figure 3, black traces, shows the across-session mean performance in decoding target location from small windows of summed spike counts for each target. Here, Target 1 (middle right) has been rotated for each session to represent the target location most preferred by the neural population recorded on that day (as determined by microstimulation; see Materials and Methods). By aligning all sessions according to their preferred target location, we can better appreciate any change in decoding target location as a function of the proximity of a target to the preferred target. In other words, Targets 2 and 8 are approximately equidistant from the preferred target, while Target 5 represents the target diametrically opposite to the preferred target, one that is in the opposite hemifield.

The first, and perhaps most obvious, observation to note is that spatial information is best decoded during the neural populations’ visual and motor bursts, peaking roughly 150 ms after target onset and again around saccade onset, respectively. This aligns well with the population-averaged response during these two epochs (Fig. 2*A*). Next, and perhaps just as intuitive, is the observation that the decoding performance is best for the target in the preferred location. Equivalently, the spiking activity pattern is most distinct from other target locations when the target is presented in the preferred location (i.e., the target that evokes a maximal firing rate in response to its appearance).

Equally importantly, note that spatial information can still be decoded from targets far away from the preferred location (e.g., Targets 4–6). Despite the low firing rate modulation for these targets, the spiking activity is in fact still distinct across targets presented in this region; otherwise, the performance would remain at baseline level (here, at 0 on the *y*-axis) throughout the trial. Instead, the classification performance is well above chance level for these directions, including for the location diametrically opposite the preferred direction. This result can likely be attributed to the activity seen in individual channels when targets in this region are presented, although the direction of modulation (i.e., elevation or suppression of activity) for saccade targets in this hemifield is unique to each individual neuron and population (see example session in Fig. 2*A*). The last main observation in Figure 3, black traces, is that the decoding performance remains elevated throughout the delay period, in the time between the transient visual burst and the much-later motor burst, especially for targets in and near the preferred location. This result suggests that target location is one form of information still present during the delay period, which can be attributed to the sustained tonic activity exhibited by many SC neurons following the end of the transient visual response.

**Figure 4.**
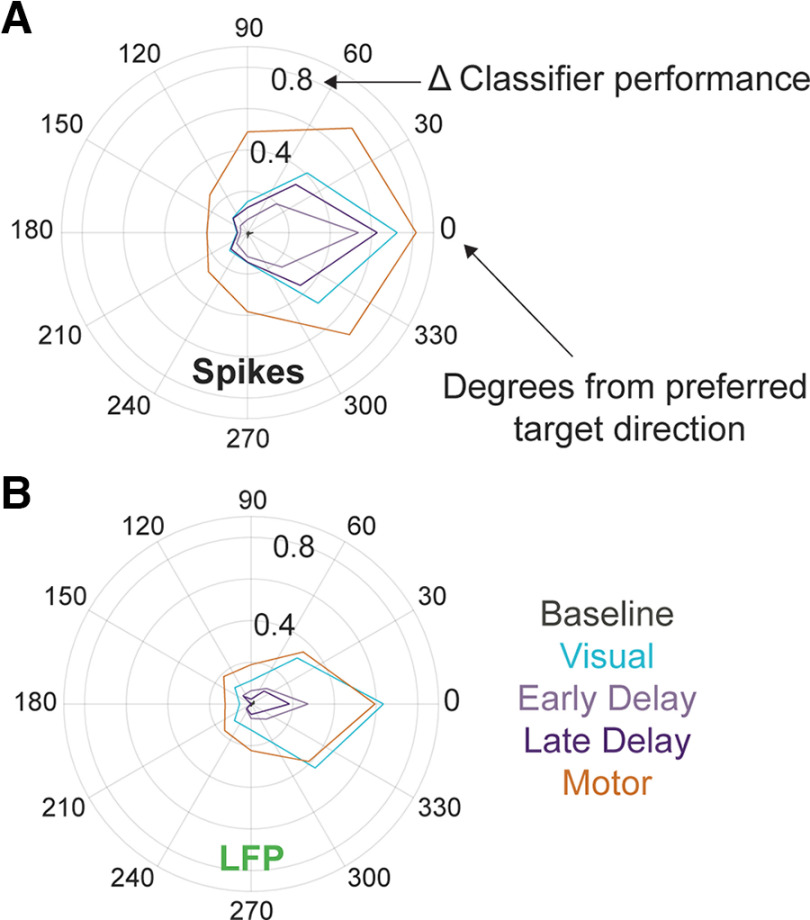
Spread of spatial direction discrimination across broad visual space. Summary polar plots of mean across-session classifier performance distribution across target directions during each epoch as defined in Materials and Methods for spiking (***A***) and LFP (***B***) activity. Spatial tuning of spiking activity is broader in the motor epoch than any other epoch. For LFPs, decoding performance is lower during the delay period but is comparable between the visual and motor epochs.

Next, we applied a classification algorithm to the LFPs recorded simultaneously across many channels. Figure 3, green traces, shows the across-session mean performance when decoding target location from small windows of averaged LFP voltage signals. A decoding performance comparable to the spike count-based classifier was found during the visual epoch. However, in contrast to the spiking activity-based classification, the ability to decode spatial location from LFPs during the delay period is much more constrained to the preferred target direction. This tuning once again becomes broader during the motor epoch, although the extent of spatial information does not expand past that observed during the visual epoch as it does in the spike-based classifier. A summary of the spread of performance for the spike count and LFP classifiers during five key epochs—baseline, visual, early delay, late delay, and motor—is presented in Figure 4.

**Figure 5.**
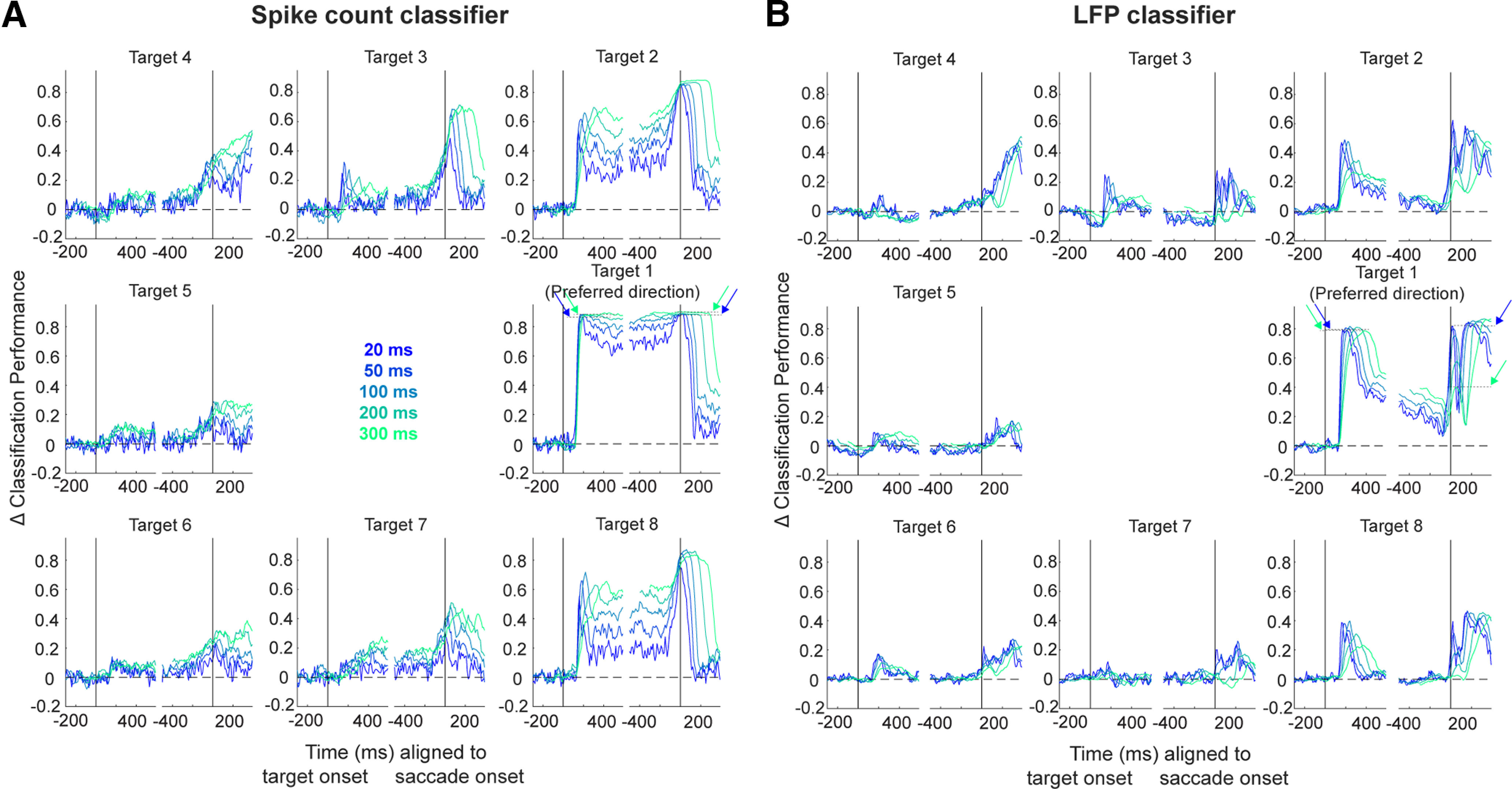
Linear discriminant classification of spiking and LFP activity: systematic variation of bin width. ***A***, Classifiers were trained and tested on summed spike counts during windows of lengths ranging from 20 to 300 ms. Average performance values over 50 bootstrapping iterations for each target direction and each window length condition is plotted using the same conventions as Figure 3 for one example session. Again, values are plotted aligned to the end of each window; therefore, each condition peaks in classification performance at different times, but this is not the comparison of interest. Spike count-based classification is largely robust to window size during the transient visual and motor epochs (as indicated by the dark blue and light green arrows at Target 1) but performance increases with increasing window sizes during the delay period. ***B***, As in ***A*** but for average LFP voltage on each channel during windows of varying lengths. A decrease in performance with increasing window lengths can be seen during the motor epoch (indicated by dark blue and light green arrows at Target 1), but the opposite effect can be seen during the delay period.

We next determined whether these observations were robust to the size of the window used to classify the target location. Therefore, we systematically varied the bin width of summed spike counts used to train and test the classifier from very small (20 ms) to very large (300 ms), and the across-session mean performance for each bin width is shown in Figure 5*A* for one example session. Indeed, varying the bin width did not qualitatively change the conclusions drawn above. Instead, the spatial location decoding performance gradually increased as bin width increased, plateauing around the 100-ms window length. In other words, using summed spiking activity from time ranges longer than 100 ms did not improve the classifier performance, from which we infer that information about spatial location is encoded maximally in short-to-medium-length periods of spiking. In contrast, when the LFP signal is averaged across windows ranging from 20 to 300 ms in length, as shown in Figure 5*B*, we see that the maximum performance is reached when the window length is the shortest during the visual and motor epochs (see dark blue and light green arrows for Target 1). This short optimal window length suggests that spatial information is encoded maximally in short periods of time during these transient epochs, unlike that observed in the spike-based classifier. However, just as with spiking activity, spatial information seems to be maximally encoded on a longer time scale during the delay period.

**Figure 6.**
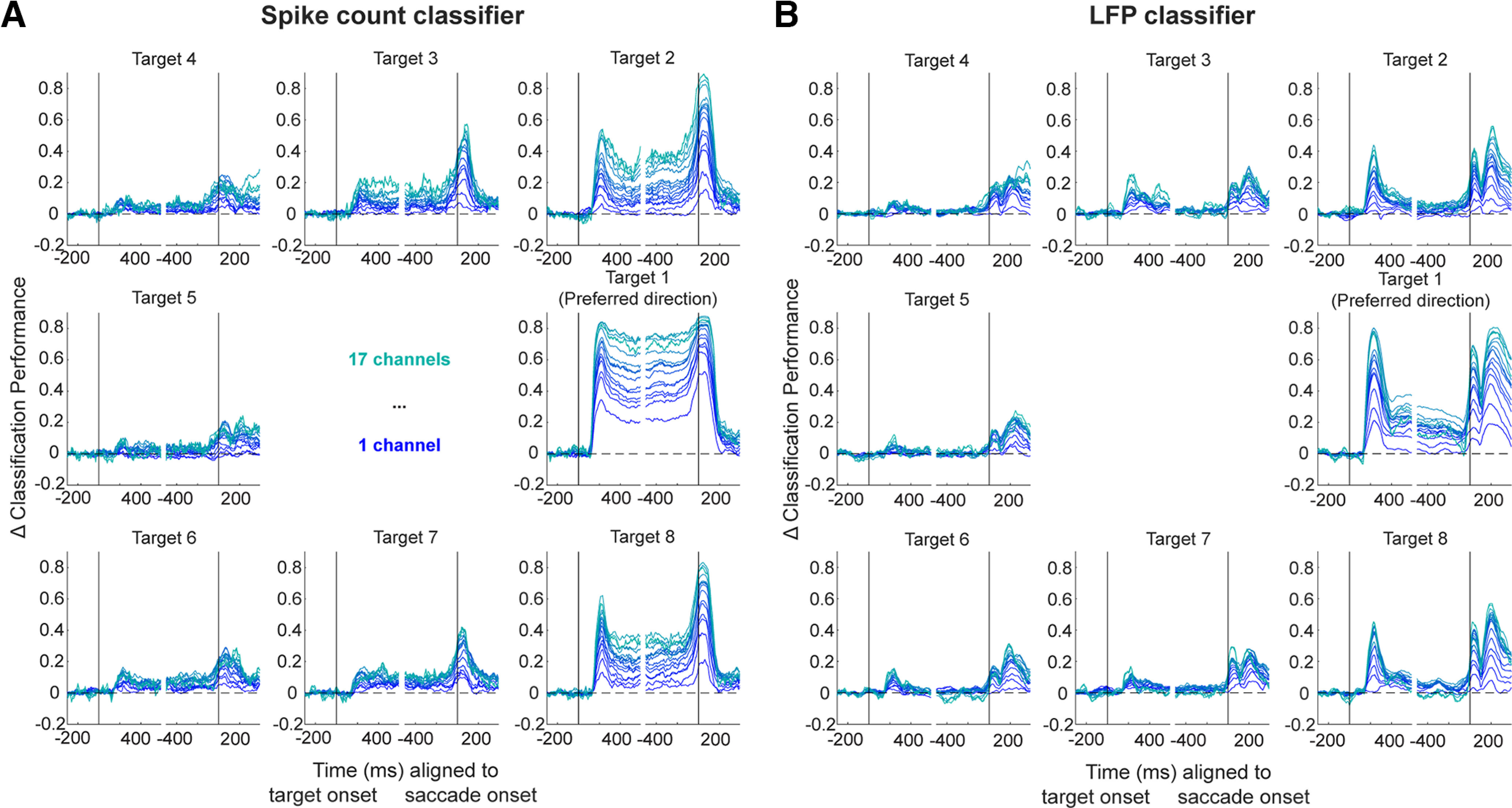
Linear discriminant classification of spiking and LFP activity: systematic variation of population size. ***A***, Classifiers were trained and tested on summed spike counts during 100-ms windows with randomly selected population sizes ranging from 1 to 17 channels. Average performance over 50 bootstrapping iterations for each target direction and each population size condition are plotted using the same conventions as Figure 3 for one example session. As population size increases, classification performance increases in a corresponding fashion. ***B***, As in ***A*** but for classifiers based on average LFP voltage across a varied number of included channels (matched to the channels included in the spike count classifiers). LFP-based classifier performance also increases systematically as a function of population size.

**Figure 7.**
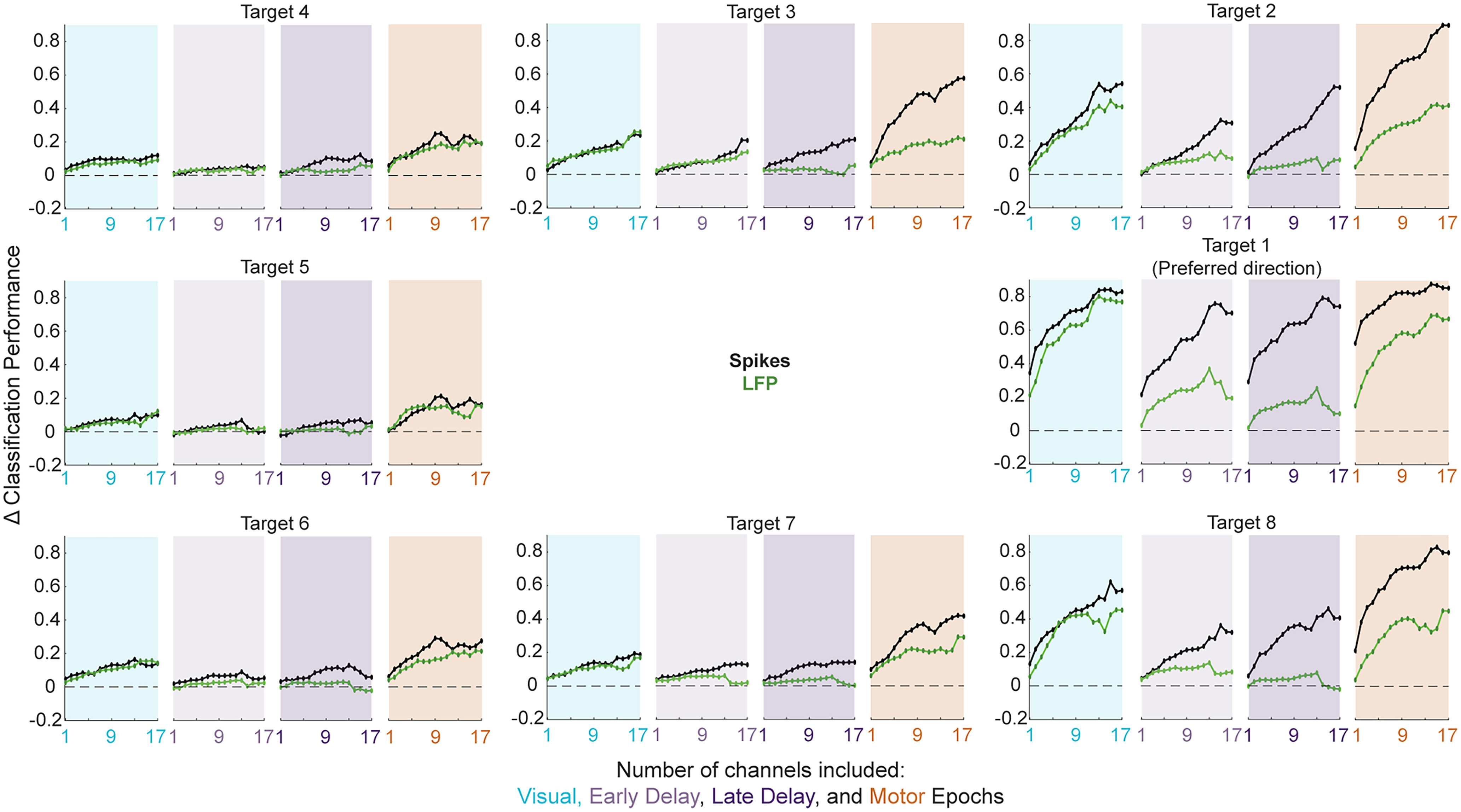
Classification performance as a function of population size during four key epochs. The performance of spike count (black traces) and LFP (green traces) classifiers was evaluated through a systematic variation of population size (Fig. 6). Here, the across-session average peak classification performance for each target during the visual (blue panels), early delay (light purple), late delay (dark purple), and motor (orange panels) epochs is plotted as a function of the number of channels included (from 1 to U; see Materials and Methods). During both the visual and motor epochs, increasing population size leads to a corresponding increase in direction discriminability, even for targets in the hemifield opposite the preferred direction. For spike count-based classifiers, performance in the delay period follows the same trend, whereas less consistency is observed in the performance of LFP-based classifiers during these epochs.

Perhaps most importantly, we asked whether the same level decoding performance could be achieved by only selecting a random channel as if using a traditional single electrode or a subset of channels to artificially decrease population size. Figure 6 shows the result of this systematic variation in population size from one channel up to U channels, which is equivalent to the number of functional channels recorded in a given session. For both spike count and LFP classifiers, average across-session performance increases nearly monotonically as population size increases. A breakdown of this trend during four key epochs (visual, early delay, late delay, and motor) can be seen in Figure 7. Hence, it is effective to decode the spatial parameter of direction from seemingly homogenous neural populations in the SC.

We also repeated the classification process after dividing each session’s channels into three subpopulations based on each channel’s relative firing rate in the visual and motor epochs (through a standard visuomotor index). As expected, the subpopulations with the highest firing rates during the visual epoch (presumably located in the more superficial SC layers) yielded the highest classifier performance of all subpopulations during the visual epoch (observations not shown). In a similar fashion, the more motoric populations (likely located in the deeper SC layers) led to the highest performance during the motor epoch (observations not shown). However, we did not aim to isolate purely visual or purely motor neurons when collecting data and consequently could not fully tease apart the relationship between neuron subtype and temporal dynamics of classifier performance.

**Figure 8.**
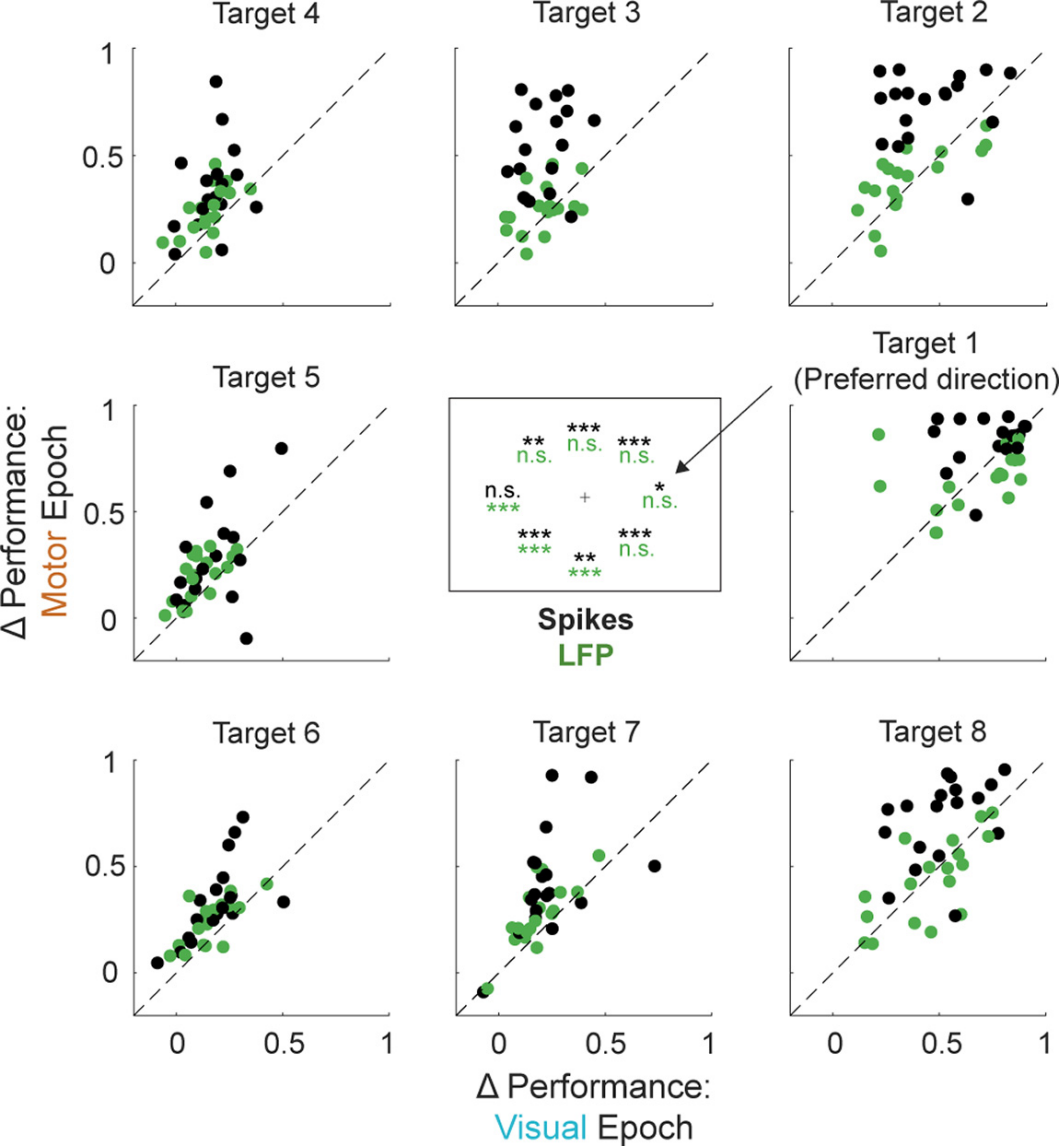
Comparison of direction encoding during the visual and motor epochs for each target. Peak decoding performance in the visual (*x*-axis) versus motor (*y*-axis) epoch as defined in Materials and Methods for each target. Spike-based classifiers are indicated in black and LFP-based classifiers are indicated in green. Each session (*N* = 18) contributes two points to each of the eight target subplots: one for spiking activity and another for LFP activity. Inset, Significant (paired *t* test) differences in performance level during the visual and motor epochs for each target are represented, with *p* < 0.05 indicated by a single asterisk, *p* < 0.01 by double asterisks, and *p* < 0.001 by triple asterisks. For spike-based classifiers, the performance is significantly different between epochs for all targets but one. For LFP-based classifiers, only targets far from the preferred direction have significantly different encoding across epochs.

Last, we quantitatively compared the spatial encoding properties across epochs and signal modalities—first for each individual target direction and then integrated across all eight target directions. Figure 8 breaks down the classification performance during the visual epoch versus the motor epoch independently for each target and signal modality. Each point corresponds to the peak decoding performance during the visual and motor epochs for a single session and target direction. We tested whether for each target and modality the performance was significantly different between the two epochs through a paired *t* test, which is shown in the inset of Figure 8. The spike-based classifier produced consistently higher performance in the motor epoch than in the visual epoch for nearly all target directions regardless of the angular distance from the preferred location. On the contrary, the LFP-based classifier only displayed significantly different performance between the visual and motor epochs for target directions far from the preferred direction.

**Figure 9.**
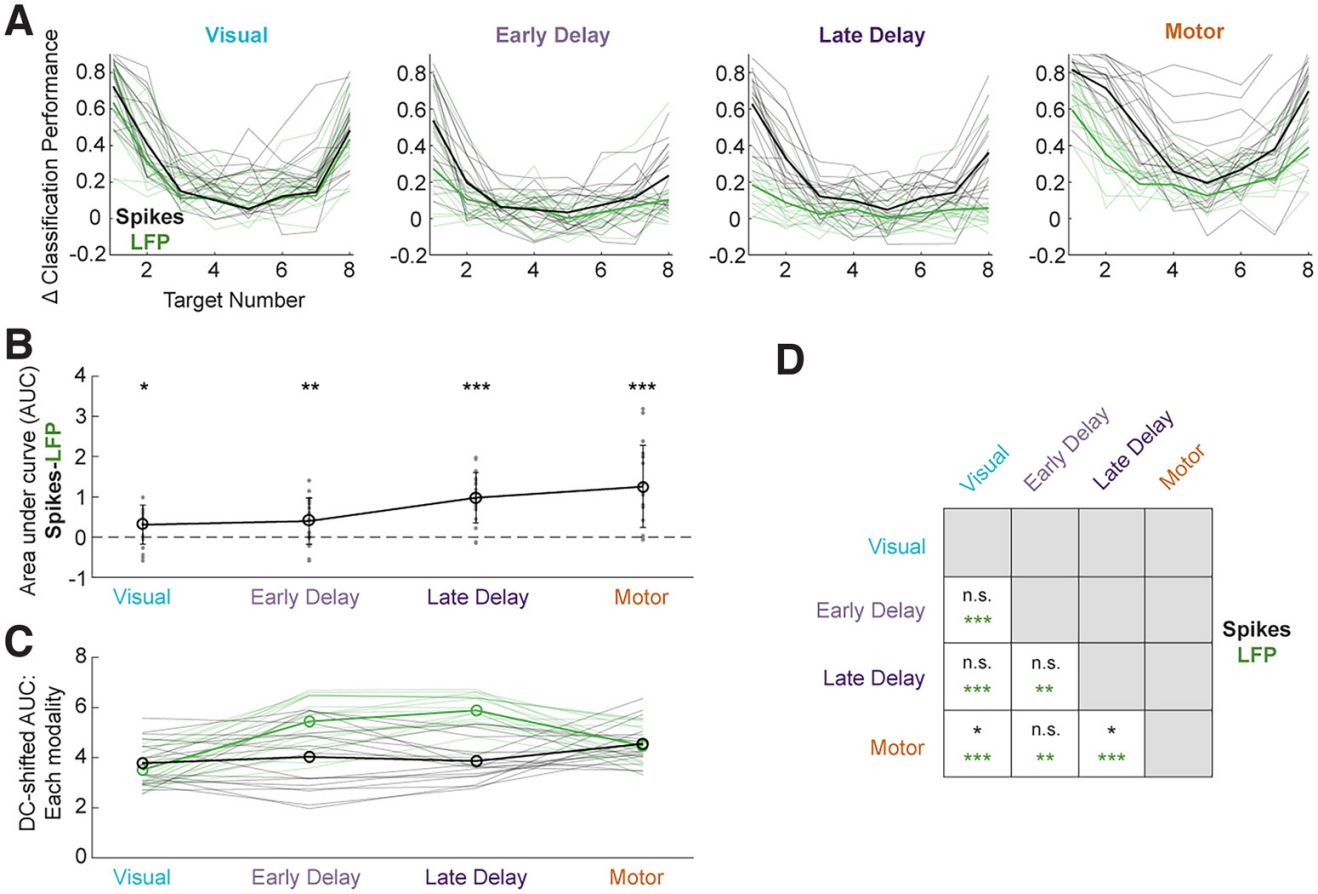
Comparison of spatial encoding properties of spiking and LFP activity across epochs. ***A***, Baseline-shifted classification performance on spiking (black) and LFP (green) activity during each of the four main epochs (as defined in Materials and Methods) for each target aligned to the preferred direction of the population. Mean across sessions (bold lines) as well as each session’s individual tuning curve (*N* = 18, thin lines) are shown. Session-averaged traces are the same as the data shown in Figure 4. ***B***, Differences in the amount of spatial information encoded between two signal modalities. Trapezoidal area under each observed tuning curve (AUC) shown in ***A*** was computed, and the LFP classifiers’ AUCs were subtracted from the spike count classifiers’ AUCs in a pairwise fashion for each session and epoch. The across-session mean difference in AUC between the two modalities (bold line) and individual session values (gray points) are plotted. Significant differences between spiking and LFP classifier distributions are shown with asterisks at the α = 0.05 significance level (paired *t* test; *p* < 0.05 is indicated by a single, *p* < 0.01 double, and *p* < 0.001 triple asterisk). From the visual epoch, the encoding of spatial information is significantly different between spiking and LFP signals. ***C***, The AUC during each epoch for each session (thin lines) along with the across-session mean AUC (bold lines) were computed after shifting each population’s decoding values such that the decoding performance was 1 for the target in the preferred direction (i.e., Target 1). This measure allows for a fair comparison of breadth of information across epochs. ***D***, Grid of statistical differences (paired *t* test) in tuning width across pairs of epochs computed separately for each signal modality. For spiking activity, the tuning width is only significantly different between the visual and motor epochs and between the late delay and motor epochs. For LFPs, the tuning width is significantly different across all epochs.

To summarize both the amount and the spatial extent, or breadth, of information across all targets, we computed an area under the curve (AUC) of decoding performance separately for each epoch and signal modality. Figure 9*A* shows the decoding performance across targets during four key epochs (see Materials and Methods for definitions) for the spike-based classifier in black and the LFP-based classifier in green. The session-averaged traces are comparable to data shown in the polar plots of Figure 4. To quantify the total amount of information across all targets, we first computed in each epoch independently the trapezoidal area under the session-averaged decoding performance trace. The pairwise difference in AUC between the two signal modalities is plotted in Figure 9*B*. Beginning in the visual epoch, the amount of spatial information is significantly different between spikes and LFPs (paired *t* test), and this separation persists throughout the time course of the trial. Then, to obtain a measure of the spatial extent of classification performance—that is, the narrowness or breadth of ability to characterize neural activity across the full range of target directions—we shifted each population’s decoding values such that the decoding performance was 1 for the target in the preferred direction (i.e., Target 1) before taking the area under the tuning curve. This provides a means of normalization across epochs so that any uniform shifts in decoding performance across all targets from one epoch to another do not impact this measure. The normalized AUC for each signal modality for each epoch is shown in Figure 9*C*. Statistical testing (Fig. 9*D*) revealed that, for the spike-based classifier, the normalized AUC is only significantly different between the visual and motor epochs and between the late delay and motor epochs. For the LFP-based classifier, the tuning width is significantly different across all epochs, indicating a dynamic shift in spatial encoding across epochs.

## Discussion

In this study, we investigated the spatial discrimination properties of spiking activity and LFP signals in the SC, an oculomotor structure critical for the transformation of sensory input into motor commands. The combination of the anatomic organization of the SC and the typical electrophysiological approach lends itself to recording neural activity within a narrow column along the dorsoventral axis. Neurons within this track have largely similar preferred saccade directions as well as largely similar preferred visual target eccentricities (Gandhi and Katnani, 2011). We showed that despite this homogeneity, classification algorithms operating on the active populations can differentiate among a wide range of directions. This population-level viewpoint provides insights into the spatial extent of direction tuning that can be decoded from neurons along the dorsoventral axis of the SC that through single unit studies was thought to be essentially nonexistent for all visual angles except those close to the preferred direction.

For each short sliding window along the timeline of a delayed saccade task, a simple linear classifier was trained offline to categorize either spiking or LFP activity as belonging to one of eight directions. By evaluating the amount of change in classification performance above baseline, we obtained a singular measure of spatial information across the channels on which task-related activity was recorded. Such offline decoding algorithms have been used to characterize the spatial encoding properties of spiking activity (Ohmae et al., 2015; Boulay et al., 2016; Khanna et al., 2020) and LFP signals (Tremblay et al., 2015) in cortical oculomotor areas. Implementing classifiers to link neural activity to a behavioral phenomenon is beneficial because they provide a quantitative, comprehensive measurement of information encoding in neural populations (Glaser et al., 2020). Of note, we do not claim that the encoded information at any time represents a particular feature such as sensation, motor preparation, or motor initiation. Instead, we simply characterize the amount of information about direction present in the population throughout the timeline of sensorimotor integration. The end position of the saccade had to be within 2° of the target position to count as a correct trial, which is a negligible displacement compared with the 45° angular distance between each pair of the eight targets used as the categories for classification. Thus, we have referred to the encoded target direction and saccade direction synonymously. However, a fine-scale characterization of the time points at which SC neurons encode spatial parameters in target-centered and gaze-centered coordinates has been reported previously (Lee and Groh, 2012; Sadeh et al., 2020; Sajad et al., 2020).

Prior studies have compared the visual receptive fields of oculomotor neurons to their movement fields (equivalently, their spatial tuning properties during the respective visual and movement epochs). In cortical areas such as the FEF, the preferred target direction of individual neurons tends to be consistent between the visual and motor epochs (Khanna et al., 2020). The visual receptive fields of SC neurons have also been shown to largely overlap with their movement fields (Wurtz and Goldberg, 1972; Anderson et al., 1998), but also see Wurtz and Goldberg (1972) and Marino et al. (2008) for exceptions. Our results conform with these previous findings. When comparing the visual and motor epochs within the spiking modality, we observed that the width of discriminability across all target directions is significantly broader in the motor epoch than in the visual epoch (see Fig. 9*D*).

Of much recent interest in the neuroscience community are the questions of what and how much information about various behavioral phenomena is contained in LFP signals—questions that have elicited studies on reach kinematic encoding by LFPs in primary motor cortex (Perel et al., 2015), attention in visual cortex (Prakash et al., 2021), route selection in hippocampus (Cheng et al., 2021), and grasping postures in anterior intraparietal cortex (Lehmann and Scherberger, 2015), among others. When comparing spatial encoding properties across the two simultaneously recorded signal modalities in this study, we found that the amount of spatial information present in spiking activity and LFPs diverged beginning in the visual epoch, with the spike-based classifier consistently better at decoding target location (see Fig. 9*B*). Both signal modalities displayed similar breadth of spatial discrimination during the visual and motor epochs; in other words, the spatial extent of decoding performance across the eight targets was comparable between spike-based and LFP-based classifiers during the visual response period and motor initiation period (considered independently, see Fig. 9*D*). During the intervening delay period, the spike-based classifier performance remained high, but LFP-based performance dropped to near baseline levels for all targets except the target closest to the neural population’s preferred direction. Thus, the encoding of direction is dynamic across epochs and signal modalities in the SC. Why might there be less information about target direction contained in LFP signals? For one, we did not arrange the presented targets according to the direction that elicited the maximum LFP deflection but rather according to the direction of the saccade elicited by microstimulation. Future experiments could elucidate the maximum amount of information encoded in LFP signals by rotating target placement to best align with the LFP preferred direction.

It is possible that the radially equidistant target angles we presented did not elicit comparable firing rate conditions as schematized in Figure 1. For instance, if we entertain the notion that the SC map should be updated to include an overrepresentation of the upper visual field (Hafed and Chen, 2016), two equidistant target directions may yield imbalanced activity at the recorded location and consequently lead to a higher level of spatial discriminability. However, it is impossible to create a paradigm in which two target conditions elicit near-identical activity at the recorded location on the SC map, especially when recording from many neurons that all have slightly different preferred directions. Still, the trial-to-trial variability in firing rates and/or voltage values should obscure direction discriminability as long as these values are somewhat comparable between equidistant target directions (e.g., ±45°, ±90°, and ±135° as is the case in our experimental setup). This obfuscation should be most apparent for target directions in the opposite hemifield of the preferred direction, where activity across all channels is minimal. We see this lack of discriminability for single neurons, but this disappears as the population size is increased (Fig. 7). The above-chance direction discriminability for targets in the hemifield opposite the preferred direction is intriguing; perhaps there is even more cross-SC interaction during sensorimotor integration than previously understood.

We suggest that the SC is a suitable candidate for brain-computer interface (BCI) applications, especially in BCIs implemented to address fundamental neuroscience questions (Sadtler et al., 2014). Although the vast majority of prior work that implements closed loop control of a computer cursor or robot arm has decoded neural activity from skeletomotor structures, a few groups have ventured into the oculomotor domain and demonstrated that volitional control of neural activity is possible in these areas (Schafer and Moore, 2011; Graf and Andersen, 2014; Jia et al., 2017) as well as in wholly nonmotor areas (e.g., primary visual cortex; Neely et al., 2018). We foresee two possible limitations to using SC neurons or LFPs to decode intended saccade direction. First, the SC is a deep brain structure, which imposes a constraint on the number of recordable electrode sites. Cortical arrays fit electrode sites on the scale of hundreds, while laminar probes suitable for deep brain recording only allow for contacts on the order of tens. This is the likely reason that prior implementations of oculomotor BCIs have targeted cortical regions such as the lateral intraparietal area (LIP), frontal eye fields (FEF), and supplementary eye fields (SEF). However, advances in technology (e.g., Neuropixels) may soon negate this limitation. Second, the organization of neurons within a column along the dorsoventral axis results in neural populations with largely the same tuning properties (Gandhi and Katnani, 2011). This homogeneity theoretically reduces the spatial extent of decoding capability to targets far from the preferred target location, although we surprisingly observed that this is not the case; in fact, even targets in the diametrically opposite location of the preferred direction have above-chance decoding performance during the putatively preparatory delay period when the classifier is based on spiking activity (Fig. 3, black traces). Nonetheless, a neural population with more varied preferred directions would maximize the spatial extent of high decoding performance. Recording from the FEF, a cortical oculomotor area, yields much more heterogeneity in directional tuning across electrode depth (Bruce et al., 1985), although because of its position in the bank of the arcuate sulcus the first limitation would still apply. Therefore, we are eager for the field to recognize the potential the SC has for brain-computer interface applications.

## Synthesis

Reviewing Editor: David Schoppik, New York University Decisions are customarily a result of the Reviewing Editor and the peer reviewers coming together and discussing their recommendations until a consensus is reached. When revisions are invited, a fact-based synthesis statement explaining their decision and outlining what is needed to prepare a revision will be listed below. The following reviewer(s) agreed to reveal their identity: NONE.

Thank you for your patience. One reviewer and I evaluated the manuscript. We found it to be largely lucid, thoughtful, and a valuable contribution to the literature. Below are a number of suggestions for clarifications/additional analyses we thought might strengthen the manuscript.

1. The manuscript shows how classifier performance varies with the number of channels. Have the authors considered using a similar analysis to evaluate whether the spatial position of the channel on the electrode (i.e. whether it is more superficial/deep) impacts classifier performance? Furthermore, does that relationship depend on the time epoch being analyzed? Naively, we wondered if more superficial channels impact classifier performance more strongly early in the trial, and deep channels later on. We felt that the manuscript would be stronger for such an analysis.

2. We had a number of questions related to the methods:

How many saccades were averaged to calculate the “preferred location” (L105) for each session?

Please provide the vector of the evoked saccades. Adding a figure that indicates all 15 probe locations in the left and right SCs separately (e.g., plot the probe location on a figure like Fig. 1A left) would be ideal.

Please add stimulation conditions (frequency, polarity, duration, voltage, etc.).

How much was the vector of the evoked saccades different between most dorsal and ventral stimulations? How was the preferred vector calculated if they are largely different?

According to Fig. 1D, the probe was placed mostly in the intermediate layer. Was the probe placed like this in all 15 sessions? How did the authors decide the depth of the probe during experiments; that is, when did the authors stop driving the probe down?

Were the “functional channels” (L 110) for recording and the channels that evoked saccades by stimulation the same?

3. And similarly, a number of clarifications for the behavioral paradigm:

Does the data include only saccades from the center to the peripheral target, or does the data also include saccades from the peripheral to the center target?

In this delayed saccade task, how long was the delay? Were monkeys able to anticipate the timing?

Please clarify the criteria of the “successful trial” (L126); the value appears later in the manuscript but it would be useful to have it here as well.

How long did it take for one session? How stable was the multi-contact probe recording, and was the probe depth adjusted during the session? How long did you wait to start recording after you inserted the probe into the SC?

It would be helpful for comparative purposes to run standard statistical tests first (for example, ANOVA) to describe features of the data like tuning strength.

The analysis time window was 100ms but it was 200ms only for the baseline period (L 148), should this be 100ms?
